# Adsorption/Desorption of Cationic-Hydrophobic Peptides on Zwitterionic Lipid Bilayer Is Associated with the Possibility of Proton Transfer

**DOI:** 10.3390/antibiotics12071216

**Published:** 2023-07-21

**Authors:** Lea Pašalić, Andreja Jakas, Barbara Pem, Danijela Bakarić

**Affiliations:** Division of Organic Chemistry and Biochemistry, Ruđer Bošković Institute, Bijenička Cesta 54, 10000 Zagreb, Croatia

**Keywords:** cell-penetrating peptides R5F2 and K5F2, large unilamellar liposomes (LUV), 1,2-dipalmitoyl-*sn*-glycero-3-phosphocholine (DPPC), adsorption mechanism, FTIR and UV-Vis spectroscopy, MD simulations

## Abstract

Cell-penetrating peptides (CPPs) are short peptides built up from dominantly cationic and hydrophobic amino acid residues with a distinguished ability to pass through the cell membrane. Due to the possibility of linking and delivering the appropriate cargo at the desired location, CPPs are considered an economic and less invasive alternative to antibiotics. Besides knowing that their membrane passage mechanism is a complex function of CPP chemical composition, the ionic strength of the solution, and the membrane composition, all other details on how they penetrate cell membranes are rather vague. The aim of this study is to elucidate the ad(de)sorption of arginine-/lysine- and phenylalanine-rich peptides on a lipid membrane composed of 1,2-dipalmitoyl-*sn*-glycero-3-phosphocholine (DPPC) lipids. DSC and temperature-dependent UV-Vis measurements confirmed the impact of the adsorbed peptides on thermotropic properties of DPPC, but in an inconclusive way. On the other hand, FTIR spectra acquired at 30 °C and 50 °C (when DPPC lipids are found in the gel and fluid phase, respectively) unambiguously confirmed the proton transfer between particular titratable functional groups of R5F2/K5F2 that highly depend on their immediate surroundings (DPPC or a phosphate buffer). Molecular dynamic simulations showed that both peptides may adsorb onto the bilayer, but K5F2 desorbs more easily and favors the solvent, while R5F2 remains attached. The results obtained in this work highlight the importance of proton transfer in the design of CPPs with their desired cargo, as its charge and composition dictates the possibility of entering the cell.

## 1. Introduction 

One of the turning points in modern medicine was the discovery of antibiotics, medicines prepared with the aim of either killing bacterial cells or preventing their further colonization of the host cells [1,2,3]. Thanks to the use of antibiotics, people no longer died from seemingly harmless bacterial infections, and the quality and duration of human life was significantly improved and extended. However, as bacterial cells are able to mutate in a relatively short time frame and gradually develop resistance to existing antibiotics, one of the contemporary problems in the modern treatment of bacterial infections is to prevent the emergence of antibiotic-resistant bacterial strains [4]. Although the synthesis of new classes of antibiotics may seem like a possible solution, one must ignore the fact that antibiotics themselves are more or less harmful to human cells, and also that their laboratory synthesis, characterization and purification is ultimately a very expensive process. Therefore, the synthesis of significantly simpler components that are able to penetrate the bacterial membrane and thus directly or indirectly prevent the proliferation of bacterial cells and/or their destruction is recognized as an alternative to the conventional use of antibiotics.

The direct and indirect destruction of bacterial cells can be achieved through the usage of antimicrobial and cell-penetrating peptides (AMPs and CPPs) [4,5,6,7,8,9] that contain a considerable fraction of cationic amino acids, especially Arginine (Arg or R) and Lysine (Lys or K), and hydrophobic ones. The interaction of AMPs with plasma membranes generally encompasses the electrostatic attraction of peptide cationic residues and plasma membrane anionic lipids, which is accompanied by the conformation change of hydrophobic peptide parts, ultimately resulting in their anchoring to the membrane surface [8,10]. A direct disruption of the bacterial cell membrane or the binding to the intracellular species essential for bacterial cell proliferation and survival stands as a crucial difference between AMPs and CPPs. The latter are, on the other hand, designed with the aim to carry and deliver a specific molecule able to destroy the bacterial cell that cannot pass the bacterial cell membrane on its own. The penetration mechanism of CPPs into the cell varies with peptide composition, with the cargo attached or associated with it, as well as with the composition of cell membrane. Despite the existence of a general consensus that CPPs translocate into the cell either by a direct penetration or by endocytosis [11,12,13], the complexity of CPP internalization is mirrored already in the internalization of peptides as simple as R9 and K9. As Robison et al. reported, the adsorption of R9 on zwitterionic lipid bilayers composed from 1-palmitoyl-2-oleoyl-*sn*-glycero-3-phosphocholine (POPC) containing a variable amount of 1-palmitoyl-2-oleoyl-*sn*-glycero-3-phospho-(1′-rac-glycerol) (sodium salt) (POPG) is by far more efficient for R9 than for K9 [14]. Additionally, it appears that the adsorption of R9/K9 highly depends on peptide concentration [15,16], ionic strength and the composition of the aqueous surroundings [17] as well as on the composition of the lipid membrane [18]. 

In addition to thorough experimental and computational studies of the adsorption of R9/K9 peptides on model lipid membranes, it is crucial to mention the study in which Kamat et al. induced the localization of RNA molecules compartmentalized in POPC liposomes by the adsorption of short cationic-hydrophobic peptides on the outer membrane leaflet of the same liposome [19]. Although the primary goal in the mentioned study was to examine the catalytic potential of the lipid membrane surface and its possible role in the origin of primitive cell development [19,20], one of the outstanding findings that emerged from that work is the discovery that some of the explored peptides can traverse through the lipid membrane. As the potency of peptides containing phenylalanine (Phe or F) and R-residues appeared to be among the highest in the explored set, it seemed self-evident that the passage mechanism of such peptides should be investigated more thoroughly. Apart from the knowledge that cationic amino acid residues will have electrostatic interactions with titratable functional groups of lipids, and hydrophobic with non-polar ones, every other detail of the interaction of prepared peptides and zwitterionic lipid membranes is still unavailable. 

**Figure 1:**
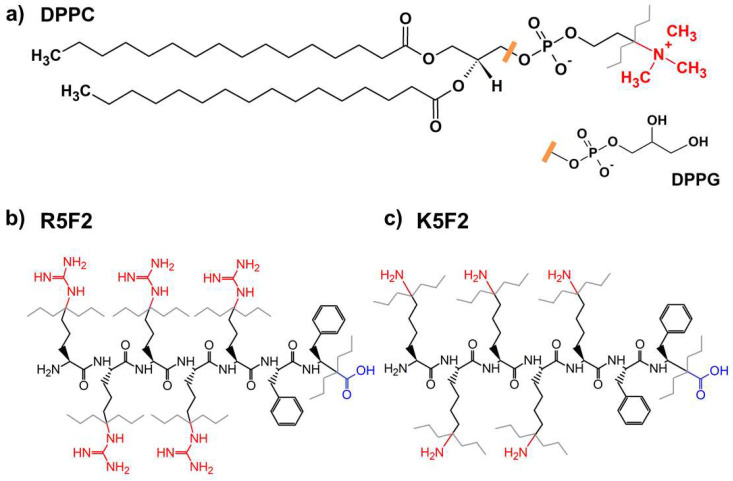
Structural formulas of: (**a**) DPPC (with DPPG headgroup); (**b**) R5F2; (**c**) K5F2. Color and gray zig-zag lines denote functional groups that may participate in proton-exchange (see below).

As membranes of both prokaryotes and eukaryotes are constituted from a mixture of zwitterionic and anionic lipids, the reconstruction of the mechanism of investigated CPP cell entrance requires the study of their interaction with both zwitterionic and anionic lipids, as well as with their mixture. Thus, the goal of the research presented in this manuscript is to elucidate the interaction of the chosen CPPs with large unilamellar liposomes (LUVs) constituted from 1,2-dipalmitoyl-*sn*-glycero-3-phosphocholine (DPPC) lipids (Figure 1). Since R5F2 is among the most potent CPP species, we sought to elucidate not only their adsorption mechanism on the DPPC lipid bilayer, but also the adsorption mechanism of the K5F2 peptide (Figure 1). Although the latter peptide was not the subject of research in the context of CPPs, the general importance of K-based peptides is not only evident from the cited literature, but also from our previous study of the adsorption of guanidinium (Gdm^+^) and ammonium (NH_4_^+^) cations, as model amino acids R and K, on zwitterionic and anionic lipid bilayers, which showed that proton-transfer is one of the key differences in their adsorption mechanism [21]. Regarding the choice of lipids, we focused on DPPC, which at experimentally attainable temperatures can be found in the gel phase (L_β′_), the ripple phase (P_β′_) and the fluid phase (L_α_). Although from the biological point of view the importance of DPPC rises up only as a lung surfactant [22,23], the adsorption of CPPs on the surface of DPPC lipid membranes should not differ too much from those of POPC. Further, certain differences in the temperatures of achievable phase transitions (pretransition temperature (*T*_p_) for L_β′_→P_β′_ and main phase transition temperature (*T*_m_) for P_β′_→L_α_) [24,25,26] indirectly enable the assessment of the influence of adsorption on the thermotropic properties of DPPC lipids. In order to prevent premature lamellarization and aggregation of DPPC LUVs, a small amount (5% mole ratio) of 1,2-dipalmitoyl-*sn*-glycero-3-phospho-(1′-rac-glycerol) (sodium salt) (DPPG) is incorporated in LUVs [27] (Figure 1). Finally, from the results obtained we were able to identify crucial differences in the adsorption mechanism of R5F2 and K5F2 peptides in terms of proton transfer that inevitably have to be considered when one designs peptides that should not just pass the cell membrane but also carry some (un)charged cargo whose sole purpose is to act in a selective manner on bacterial and human cells. 

## 2. Experimental

### 2.1. Chemicals and Liposome Preparation

Peptides R5F2 and K5F2 were synthesized following the solid-phase peptide synthesis protocol [28] (all details regarding their preparation, purification and characterization are described in Supporting Information in Section S1). 1,2-dipalmitoyl-*sn*-glycero-3-phosphocholine (DPPC) and 1,2-dipalmitoyl-*sn*-glycero-3-phospho-(1′-rac-glycerol) sodium salts (DPPG) were purchased as white powders from Avanti Polar Lipids (≥99%). Their stock solutions in chloroform (CHCl_3_; colorless liquid, p.a., Carlo Erba) of concentration *γ*(DPPC) = 10 mg mL^−1^ (*c* = 0.0135 mol dm^−3^) and *γ*(DPPG) = 1 mg mL^−1^ (*c* = 0.001 mol dm^−3^) were prepared by dissolving 50 mg/10 mg of DPPC/DPPG in 5 mL/10 mL of CHCl_3_ that were further used for the preparation of multilamellar (MLV) DPPC + 5% DPPG liposomes. Firstly, the aqueous solution of phosphate buffer (PB) of ionic strength (*I* = 100 mmol dm^−3^) was prepared from commercially available sodium hydrogen phosphate, anhydrous (Na_2_HPO_4_, ≥99%, Kemika, Zagreb, Croatia) and sodium phosphate monobasic (NaH_2_PO_4_, p.a., Kemika) in Milli-Q water and titrated with freshly prepared sodium hydroxide solution (NaOH, T.T.T., p.a.) of ionic strength (*I*(NaOH) = 100 mmol dm^−3^) in order to reach pH ≈ 7.4. In two separate flasks, 3 mL of DPPC stock solution and 1.5 mL of DPPG stock solution were pipetted. The flask was placed on a rotary evaporator and CHCl_3_ was evaporated under the pressure of 600 to 300 mbar. After drying the obtained lipid films under an Ar stream, the films were dissolved in 6 mL of PB (*I* = 100 mmol dm^−3^), and the mass concentration of lipid was *γ*(DPPC + DPPG) = 5 mg mL^−1^ (*c* = 0.0066 mol dm^−3^). The preparation of MLVs constituted from DPPC + 5% DPPG (in the continuation of the text labelled as DPPC′) took place in 3 steps, starting with vortexing the suspensions, heating them up in a hot H_2_O bath (70 °C) and cooling them in an ice bath (~4 °C). Before adding the peptides to the lipid suspension, 10 mg of R5F2/K5F2 peptides were dissolved in 10 mL of PB (pH ≈ 7.4) to mass concentration *γ* = 1 mg mL^−1^ (*c* = 0.01 mol dm^−3^). In prepared MLVs, R5F2 and K5F2 were added at a 1:30 peptide-to-lipid molar ratio. Precisely 1486 μL of R5F2 peptide was added to one flask with DPPC′ MLVs and 1360 μL of K5F2 to another flask with MLV DPPC′ suspension. MLV suspensions with peptides were pushed at least 31 times through an Avanti^®^ Mini Extruder with a holder/heating block (50 °C) through a 100 nm size polycarbonate membrane and with the assistance of 10 mm supporting filters in order to obtain large unilamellar liposomes (LUVs) of DPPC′. Mass concentrations of LUVs DPPC′ with peptides were *γ* = 5 mg mL^−1^ for DSC and FTIR measurements, *γ* =1 mg mL^−1^ for UV-Vis measurements and *γ* = 0.05 mg mL^−1^ for DLS measurements, respectively. 

### 2.2. Dynamic Light Scattering (DLS): Measurements and Data Analysis

The size distribution of LUVs was assessed with dynamic light scattering using a photon correlation spectrophotometer equipped with a 532 nm (green) laser (Zetasizer Nano ZS, Malvern Instruments, Worcestershire, UK). The average hydrodynamic diameter (*d*_h_) was specified as the value at peak maximum of the volume distribution. The reported results correspond to the average of six measurements at 25 °C. The data processing was conducted by the Zetasizer software 802 (Malvern Instruments). The average hydrodynamic diameter values of the LUVs of DPPC′ ± R5F2/K5F2 (at *γ*/*c*(DPPC′ ± R5F2/K5F2) = 0.05 mg mL^−1^/6.8 × 10^−5^ mol dm^−3^, *c*(R5F2/K5F2) = 5.2 × 10^−5^ mol dm^−3^) were in the range and 130 nm ≤ *d*_h_ ≤ 145 nm for DPPC′, 130 nm ≤ *d*_h_ ≤ 175 nm for DPPC′ + R5F2, and 160 nm ≤ *d*_h_ ≤ 225 nm for DPPC′ + K5F2 (see Section S2 and Figure S1 in Supporting Information for more details).

### 2.3. Differential Scanning Calorimetry (DSC): Data Acquisition and Curve Analysis

**Figure 2:**
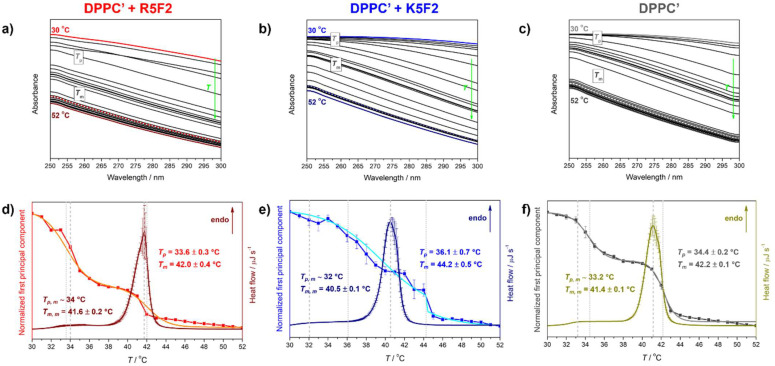
Temperature-dependent UV-Vis spectra (solid curves) and spectral profiles (dotted curves) of: (**a**) DPPC′ + R5F2; (**b**) DPPC′ + K5F2; (**c**) DPPC′. The spectra acquired at 30 °C/50 °C are highlighted (red/wine for DPPC′ + R5F2, blue/navy for DPPC′ + K5F2, gray/dark gray for DPPC′), as well as spectral profile curves (red for DPPC′ + R5F2, blue for DPPC′ + K5F2, dark gray for DPPC′); DSC curves and concentrational profiles of the first principal component accompanied with a double Boltzmann sigmoidal transition of: (**d**) DPPC′ + R5F2 (wine curve for DSC and red/orange curve for spectral projection of UV-Vis data/double Boltzmann fit); (**e**) DPPC′ + K5F2 (navy curve for DSC and blue/cyan curve for spectral projection of UV-Vis data/double Boltzmann fit); (**f**) DPPC′ (dark yellow curve for DSC and dark gray/gray curve for spectral projection of UV-Vis data/double Boltzmann fit). Phase transition temperatures are highlighted with dashed (DSC) and dotted (UV-Vis) lines and are additionally written on graphs and designated with a corresponding color.

Calorimetric experiments were conducted on a microcalorimeter Nano-DSC, TA Instruments (New Castle, DE, USA). Before starting the measurement, suspensions were held for 10 min in a degassing station. The suspensions of DPPC′ ± R5F2/K5F2 (*γ/c*(DPPC′ ± R5F2/K5F2 = 5 mg mL^−1^/0.0066 mol dm^−3^) were recorded at a scan rate of 1 °C min^−1^ in duplicates in two repeated heating–cooling cycles in a temperature range of 20–60 °C. PB, which was taken as a reference, was examined in the temperature range of 10–90 °C. The analysis of the data obtained during the thermal-history free second heating run [26,29] was preformed using the TA Instruments Nano Analyze software package. Following the subtraction of a reference DSC curve (PB) from a DSC curve of the samples (peptides/DPPC′/peptides + DPPC′) obtained from the analogous scan (2nd heating run), the resultant curves were baseline corrected and examined in the temperature region 30–52 °C. The phase transition temperatures of pretransition (*T*_p_) and the main phase transition ™ were determined from the curve maxima (*T*_p, m_ and *T*_m, m_) [30] (Figure 2). DSC curves of R5F2/K5F2 in PB were collected as well at the same conditions in order to verify that the peptides did not display any thermotropic event in the explored temperature range (Figure S2 in Supporting Information). 

### 2.4. UV-Vis Spectroscopy: Data Acquisition and Spectral Analysis

UV-Vis spectra of DPPC′ suspensions in the absence and the presence of R5F2/K5F2 (*γ*(DPPC′ ± R5F2/K5F2) = 1 mg mL^−1^) were measured on a UV-Vis spectrophotometer Thermo Scientific Nanodrop 2000 (Thermo Fischer Scientific, Waltham, MA, USA) and on a Cary 5000 UV-Vis-NIR Spectrophotometer (Agilent Technologies, Santa Clara, CA, USA) in the spectral range of 200–500 nm. The spectra of lipid suspensions were recorded at least three times (different cuvettes) in the temperature range of 30–52 °C. The spectra of PB ± R5F2/K5F2 were collected once in the same temperature range. 

Collected temperature-dependent UV-Vis spectra of DPPC′ ± R5F2/K5F2 in the spectral range 250–300 nm were smoothed (Savitzky-Golay: polynomial of a 3rd degree through 10 points [31]) (Figure 2a–c)) and subjected to multivariate curve analysis, the details of which are thoroughly described in [27,30,32,33]. Briefly, using publicly available Matlab code [34], the spectra were represented as the product (D) of the concentrational (C) and spectral (S) profile of one component that exhausts all temperature-dependent variations of obtained spectra:

D = CS^T^ + E,

where E represents the residuals unexplained by the CS^T^ product. 

The obtained concentrational profile of this one component is of double sigmoid character in all three examined systems (DPPC′ ± R5F2/K5F2) and, according to our previous studies, their inflection points perfectly coincide with the phase transition temperatures (*T*_pt_) [27,30]. Therefore, by fitting the concentration profile on a double Boltzmann fit, the values for *T*_p_ and *T*_m_ of DPPC′ (*R*^2^ = 0.998), DPPC′ + R5F2 (*R*^2^ = 0.999) and DPPC′ + K5F2 (*R*^2^ = 0.999) were obtained. 

### 2.5. FTIR ATR Spectroscopy: Data Acquisition and Spectral Analysis

FTIR ATR spectra of solutions R5F2/K5F2 in PB (*γ*/*c*(R5F2/K5F2) = 1 mg mL^−1^/0.001 mol dm^−3^) and suspensions DPPC′ ± R5F2/K5F2 (*γ*/*c*(DPPC′) = 5 mg mL^−1^/0.0066 mol dm^−3^; *γ*/*c*(R5F2/K5F2) = 1 mg mL^−1^/0.001 mol dm^−3^) were collected on an Invenio-S Bruker spectrometer equipped with the photovoltaic LN-MCT detector and BioATR unit (the spectra of solutions of R5F2/K5F2 in PB having *γ*/*c*(R5F2/K5F2) = 10 mg mL^−1^/0.01 mol dm^−3^ acquired with aim to facilitate band assignment are displayed in Supporting Information, Figures S3 and S4). The BioATR II unit (circular with radius of 2 mm having the upper ATR crystal made of silicon and the lower of ZnSe) was continuously purged with N_2_ gas connected with the external supply and temperature-controlled using a circulating water bath of the Huber Ministat 125 temperature controller. Solutions of R5F2/K5F2 and suspensions DPPC′ ± R5F2/K5F2 were pipetted directly on the ATR crystal unit in a volume of 30 μL and their spectra were acquired against air as a background. Using OPUS 8.5 SPI (20200710) software, all spectra were collected at a nominal resolution of 2 cm^−1^ and 256 scans at two different temperatures, 30 °C and 50 °C, at which DPPC′ is either in a gel (30 °C) or fluid (50 °C) phase [21]. FTIR spectra of the solutions of R5F2/K5F2 and suspensions DPPC′ ± R5F2/K5F2 were acquired three times, whereas the PB solution was measured once.

Following the subtraction of the PB spectrum from R5F2/K5F2 and DPPC′ ± R5F2/K5F2 spectra acquired at the same temperature, the obtained difference FTIR spectra were examined in the following spectral regions: (i) 3000–2820 cm^−1^, (ii) 1780–1695 cm^−1^, (iii) 1515–1360 cm^−1^, (iv) 1275–1190 cm^−1^, (v) 1130–1020 cm^−1^ and (vi) 990–940 cm^−1^, which display the bands originated from: (i) (anti)symmetric stretching of methylene moieties (ν_(a)s_CH_2_) and those of methyl moieties (ν_as_CH_3_), (ii) carbonyl stretching of glycerol backbone (νC=O), (iii) scissoring of methylene groups (γCH_2_), symmetric stretching of carboxylic groups (ν_s_COO^−^) and bending of protonated amino moiety (δNH_3_^+^) of K5F2, (iv) antisymmetric stretching of phosphate groups and glycerol moieties (ν_as_PO_2_^−^ and ν_(a)s_C−O), (v) symmetric stretching of phosphate and their neighboring C−O groups (ν_s_PO_2_^−^ and ν_s_C−O) and (vi) antisymmetric stretching/bending modes of choline moieties (ν_as_N(CH_3_)_3_^+^/δ_as_N(CH_3_)_3_^+^) (the vibrational bands assignment was made following the references [21,35,36,37,38,39,40]). After selecting the listed spectral regions, FTIR spectra in the selected spectral ranges (regions except for (i)) were smoothed (Savitzky–Golay; polynomial of a 3rd degree through 20 points [31]), baseline corrected (two points) and normalized [31]. As spectral regions (iv) and (vi) do not display considerable differences between DPPC′ + R5F2/K5F2, they are presented and briefly discussed in the Supporting Information (Figure S5).

## 3. Molecular Dynamics Simulations

Classical molecular dynamics (MD) was used to model DPPC membranes in the presence of either R5F2 or K5F2. The bilayer consisting of 192 DPPC molecules was constructed by the CHARMM-GUI Membrane Builder module [41] and solvated with 19,200 water molecules. The bilayer was modeled with only DPPC, since it was established previously that 5% DPPG does not significantly affect membrane structure or behavior [27]. The membrane was relaxed using the established CHARMM-GUI procedure. R5F2 and K5F2 peptides were modeled separately and also relaxed in water for 50 ns. Then, each peptide was placed in the simulation box containing the bilayer so that their center of mass was located >2 nm from the bilayer surface. To maintain an ionic strength comparable to the experimental conditions, NaCl was added to the concentration of 25 mM. Five additional Cl^−^ ions were added to neutralize the peptide charge. The simulations were conducted at 30 °C and 50 °C, corresponding to the gel and fluid phases of the membrane, respectively. 

All simulations were conducted in GROMACS 2020.0 software [42] using the CHARMM36m force field [43] and the TIP3P water model [44]. Following minimization, heating was conducted for 200 ps in the NVT ensemble with the V-rescale algorithm. The production run was conducted in the NpT ensemble with the Nosé–Hoover thermostat [45] (time constant: 1 ps) and the Parrinello–Rahman barostat [46] (semi-isotropic coupling, time constant: 5 ps, target pressure: 1 bar) for a total of 300 ns. The first 100 ns of production were considered an equilibration period and omitted from subsequent analysis. Three-dimensional periodic boundary conditions were used for all simulations. Short-range Coulomb interactions and van der Waals interactions were cut off at 1.2 nm with a switching function turned on after 1.0 nm. The particle mesh Ewald (PME) procedure [47] was used for handling the long-range Coulomb interactions. A LINCS algorithm constrained the bonds involving hydrogen. The time step was 2 fs.

## 4. Results and Discussion

### 4.1. Thermotropic Properties of DPPC′ ± R5F2/K5F2: DSC and UV-Vis Data

LUVs constituted from DPPC′ in the absence and presence of R5F2/K5F2 displayed somewhat opposite trends regarding the determined phase transition temperatures (*T*_pt_), i.e., *T*_p_ and *T*_m_ values determined from calorimetric (DSC) and spectrophotometric (UV-Vis) data (see Figure 2).

Unlike pure DPPC′ that exhibits pretransition (*T*_p, m_) at ~33.2 °C (DSC)/34.4 ± 0.2 °C (UV-Vis) and main phase transition (*T*_m, m_) at 41.4 ± 0.1 °C (DSC)/42.2 ± 0.1 °C (UV-Vis), the presence of R5F2/K5F2 altered these values, but not in the same direction when different techniques are compared. In particular, in DPPC′ + R5F2, the corresponding values are ~34 °C (DSC)/33.6 ± 0.3 °C (UV-Vis) and 41.6 ± 0.2 °C (DSC)/42.0 ± 0.4 °C (UV-Vis), whereas in DPPC′ + K5F2~32 °C (DSC)/36.1 ± 0.7 °C (UV-Vis) and 40.5 ± 0.1 °C (DSC)/44.2 ± 0.5 °C (UV-Vis) (see Table 1). 

Since in this case it is unclear which technique would be preferred in the interpretation of the thermotropic properties of the investigated systems, we will try to focus only on those attributes that we can unequivocally comment on. From the point of view of DSC measurements, it is certain that the adsorption of the peptide is reflected in the undulations of the lipid bilayer, and judging by the melting profile, the adsorption of the K5F2 peptide makes a greater difference than the adsorption of the R5F2 peptide (compare Figure 2d–f). Also, the concentration profile obtained by multivariate analysis of DPPC′ + K5F2 temperature-dependent UV-Vis spectra is qualitatively significantly different from those obtained for DPPC′ ± R5F2 systems as it is accompanied by greater uncertainty and displays more or less coupled pre- and main-phase transition (Figure 2d–f). A possible interpretation of this phenomenon might emerge from the K5F2 adsorption rate on the DPPC′ surface, which will be more thoroughly discussed in the following section. 

### 4.2. Molecular Properties of DPPC′ ± R5F2/K5F2: FTIR and MD Data

**Figure 3:**
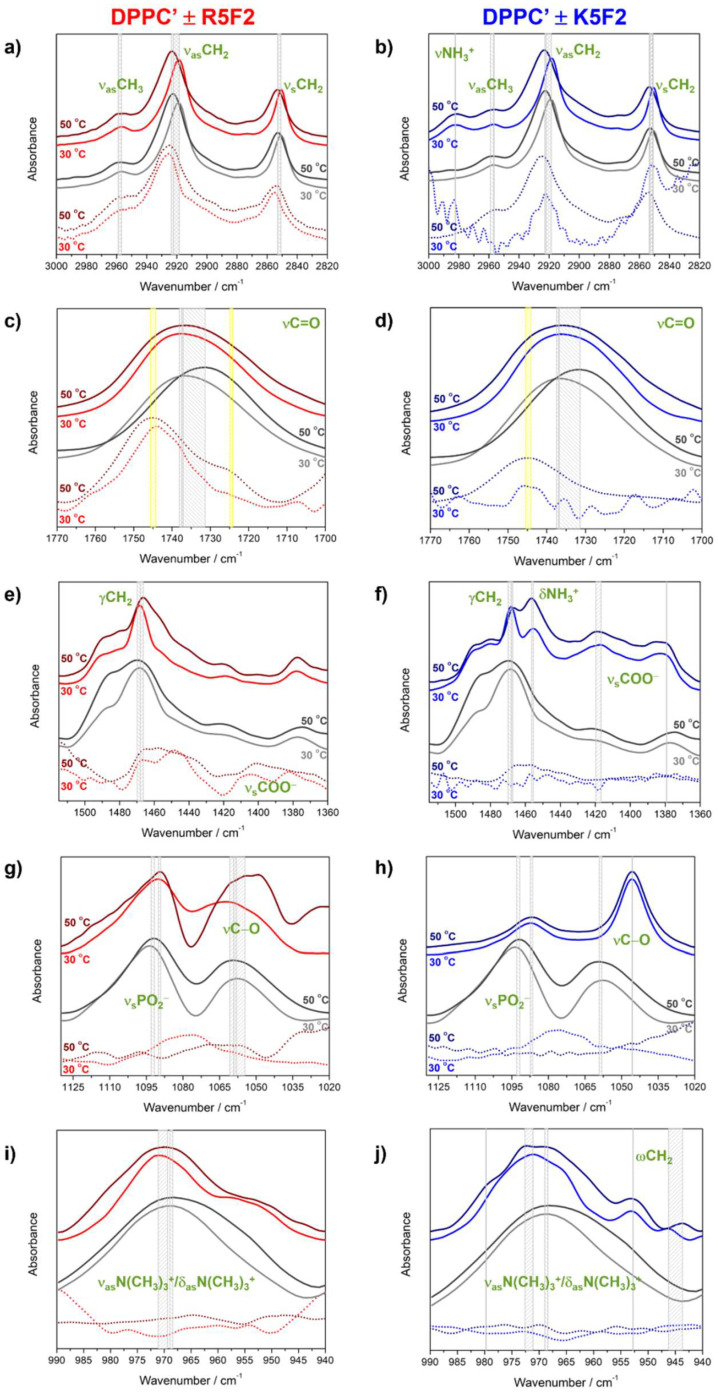
Normalized (and smoothed) FTIR spectra of DPPC′ ± R5F2/K5F2 in the following spectral ranges: (**a**,**b**) 3000–2820 cm^−1^; (**c**,**d**) 1770–1700 cm^−1^; (**e**,**f**) 1515–1360 cm^−1^; (**g**,**h**) 1130–1020 cm^−1^; (**i**,**j**) 990–940 cm^−1^. DPPC′ spectra are presented with solid gray/dark gray (30 °C/50 °C) curves, DPPC′ + R5F2 with solid red/wine (30 °C/50 °C) curves, and DPPC′ + K5F2 with solid blue/navy (30 °C/50 °C) curves. The spectra of R5F2/K5F2 are labeled with the same color as corresponding spectra of DPPC′ + R5F2/K5F2 but with dotted curves. Along with the band assignment, their displacement in DPPC′ ± R5F2/K5F2 are designated with light gray-shaded rectangles or solid light gray lines, whereas in R5F2/K5F2 spectra they are marked with yellow rectangles.

As expected, the first examined spectral region displayed a phase transition-induced high-frequency displacement of DPPC′ ν_(a)s_CH_2_ bands regardless of the presence/absence of peptides (Figure 3a,b, spectral region (i)): ν_as_CH_2_ and ν_s_CH_2_ appear at 2919 cm^−1^/2923 cm^−1^ (30 °C/50 °C) and 2850 cm^−1^/2852 cm^−1^ in DPPC′ + R5F2, at 2919 cm^−1^/2923 cm^−1^ (30 °C/50 °C) and 2851 cm^−1^/2853 cm^−1^ in DPPC′ + K5F2 and at 2919 cm^−1^/2922 cm^−1^ (30 °C/50 °C) and 2850 cm^−1^/2852 cm^−1^ in DPPC′ [21]. Besides the most distinguished lipids-originated bands, in FTIR spectra of all three examined systems there was a band originated from the antisymmetric stretching of methyl groups (ν_as_CH_3_) that displayed a small high-frequency shift upon the gel→fluid phase transition in all systems: in DPPC′ and DPPC′ + R5F2 it is 2957 cm^−1^ (30 °C)/2959 cm^−1^ (50 °C), while in DPPC′ + K5F2 it is 2957 cm^−1^ (30 °C)/2959 cm^−1^ (50 °C). Interestingly, the FTIR spectra of DPPC′ + K5F2 reveal a band at 2982 cm^−1^ (30 °C)/2982 cm^−1^ (50 °C), which is attributed to the antisymmetric stretching of NH_3_^+^ moiety (ν_as_NH_3_^+^) of Lys amino acid residues. Interestingly, the absence of a corresponding signature in FTIR spectra of K5F2, regardless of the concentration (see Figure S3b in Supporting Information), implies that the presence of DPPC′ considerably affects the interactions formed by K5F2 terminal amino moiety. 

A broad band originated from the stretching of carbonyl groups of glycerol backbone (νC=O; spectral region (ii)), Figure 3c,d)) in DPPC′ underwent a low-frequency shift due to the gel→fluid phase transition (1737 cm^−1^ at 30 °C to 1732 cm^−1^ at 50 °C), whereas the presence of peptides significantly impacted the shift magnitude; in DPPC′ + R5F2, the band maximum was displaced only for 1 cm^−1^ (1738 cm^−1^/1737 cm^−1^ at 30 °C/50 °C), whereas in DPPC′ + K5F2 there is no displacement at all (1737 cm^−1^/1737 cm^−1^ at 30 °C/50 °C). In the same spectral region, the Amide I band of R5F2 unraveled a broad envelope with two distinguished maxima (1744 cm^−1^ and 1725 cm^−1^ at 30 °C/1746 cm^−1^ and 1724 cm^−1^ at 50 °C) (Figure 3c and Figure S4 in Supporting Information), whereas for K5F2, only one weakly displaced maximum appeared upon heating (1744 cm^−1^ at 30 °C/1745 cm^−1^ at 50 °C) (Figure 3d and Figure S4 in Supporting Information). The appearances of these bands suggest that R5F2, unlike K5F2, in PB may form distinct secondary structures [48] that are differently populated as temperature changes. 

The scissoring of methylene groups (γCH_2_; spectral region (iii), Figure 3e,f), considered to be one of the most informative signals of the lateral ordering of lipid molecules [40], was displaced upon the gel→fluid phase transition from 1468 cm^−1^ (30 °C) to 1470 cm^−1^ (50 °C) in DPPC′, from 1468 cm^−1^ (30 °C) to 1466 cm^−1^ (50 °C) in DPPC′ + R5F2 and from 1468 cm^−1^ (30 °C) to 1467 cm^−1^ (50 °C) in DPPC′ + K5F2. The low-frequency displacement in the presence of R5F2/K5F2, which was opposite to the high-frequency one in DPPC′, suggests that the adsorption of peptides onto DPPC′ may cause a weakening of lateral ordering between lipid molecules. Moreover, the spectrum of DPPC′ + K5F2 in the explored spectral range exhibits features with maxima at 1455 cm^−1^/1456 cm^−1^ (30 °C/50 °C), 1417 cm^−1^/1420 cm^−1^ (30 °C /50 °C) and 1379 cm^−1^/1379 cm^−1^ (30 °C /50 °C). The first band is assigned as the bending of the protonated amino moiety (δNH_3_^+^) of terminal Lys residue, while the other two envelopes originated from the symmetric stretching of carboxylic groups (ν_s_COO^−^). Interestingly, analogous features are, at best, barely observed in the corresponding spectra of DPPC′ + R5F2, regardless of their appearance in R5F2/K5F2 in PB solutions (see Supporting Information, Figure S3e,f). This phenomenon might be related with the (partial) protonation of the COO^−^ group of R5F2 that becomes conformationally enabled upon its adsorption on the DPPC′ surface (see below for more discussion on this issue). 

The signals originated from the symmetric stretching of phosphate groups (ν_s_PO_2_^−^) and those of C−O groups (νC−O) (Figure 3g,h); spectral region (v) appears at 1094 cm^−1^ (30 °C)/1092 cm^−1^ (50 °C) and 1058 cm^−1^ (30 °C)/1059 cm^−1^ (50 °C) in DPPC′, at 1090 cm^−1^ (30 °C)/1089 cm^−1^ (50 °C) and 1061 cm^−1^ (30 °C)/1055 cm^−1^ (50 °C) in DPPC′ + R5F2 and at 1088 cm^−1^ (30 °C)/1086 cm^−1^ (50 °C) and 1046 cm^−1^ (30 °C)/1046 cm^−1^ (50 °C) in DPPC′ + K5F2, respectively. The systems containing K5F2 display differences in terms of the relative intensity ratios and the band maxima positions; opposite to the expectations (slightly stronger former band compared to the latter since the intensity of ν_s_PO_2_^−^ is usually greater than the intensity of νC−O), in DPPC′ + K5F2 the former band is much weaker than the latter, which is additionally significantly shifted to lower frequencies (Figure 3h). The most probable explanation for this appearance is that K5F2 significantly alters the vibrations of polar headgroups upon adsorption on the DPPC′ surface, probably by forming some kind of either hydrogen bonds (HBs) or salt bridges (see MD data).

Spectral regions that display the bands originated from the antisymmetric stretching of phosphate groups (ν_as_PO_2_^−^); the (anti)symmetric stretching of glycerol backbone (ν_(a)s_CO) (spectral region iv) does not differ as much in those three systems and is displayed in the Supporting Information (Figure S4).

The signals originated from the antisymmetric stretching/bending modes of choline moieties (ν_as_N(CH_3_)_3_^+^/δ_as_N(CH_3_)_3_^+^) (Figure 3i,j) appear at 969 cm^−1^ (30 °C)/968 cm^−1^ (50 °C) in DPPC′, at 976 cm^−1^ (30 °C)/969 cm^−1^ (50 °C) in DPPC′ + R5F2 and at 971 cm^−1^ (30 °C)/973 cm^−1^ (50 °C) in DPPC′ + K5F2. In the last system, there are maxima as well at 980 cm^−1^ (only at 50 °C), at 953 cm^−1^ (30 °C)/953 cm^−1^ (50 °C) and at 946 cm^−1^ (30 °C)/944 cm^−1^ (50 °C). These additional maxima might arise due to the potential interaction of choline moiety with K5F2 or the wagging of methylene groups (ωCH_2_) simply becomes pronounced in DPPC′ + K5F2 [35].

**Figure 4:**
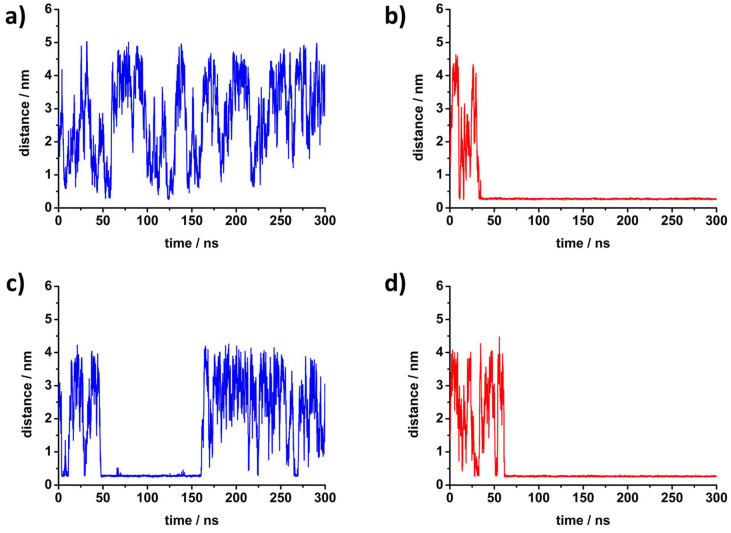
The time dependence of the minimum distance of any peptide atom from the phosphorus atom of DPPC for each investigated system in molecular dynamics simulations: (**a**) K5R2 at 30 °C, (**b**) R5F2 at 30 °C, (**c**) K5R2 at 50 °C and (**d**) R5F2 at 50 °C.

In order to further investigate peptide interactions with lipid bilayers, MD simulations were employed by placing either peptide in the simulation box with the DPPC bilayer. The peptides were initially located in the solvent phase, where they remained for the duration of minimization and heating runs. The evolution of their position during the production run is shown in Figure 4. At 30 °C, K5F2 is mostly present in the solvent, and establishes only transient contacts with the membrane, with the longest lasting less than 2 ns. At the same temperature, R5F2 adsorbs within the first 33 ns and remains attached for the remainder of the simulation. At 50 °C, both peptides adsorb during the equilibration phase, but R5F2 remains adsorbed up to the end, while K5F2 desorbs after 113 ns of contact, and establishes only transient contacts from then on. Therefore, the simulations confirm the higher affinity of Arg-based peptides for lipid membranes compared to Lys-peptides, a phenomenon that was already observed for similar systems [14,49,50]. The source of the discrepancy is due to the cationic amino acids, since those are seen in closest contact with lipid headgroups, while Phe is always further away. This is noted from the number of density functions (Figure S6 in Supporting Information) that show Phe to favor being positioned towards the solvent, and Lys/Arg towards the membrane.

The evaluation of different Lys or Arg interactions with lipid headgroups was conducted by examining the radial distribution functions (RDFs) of peptides versus the lipid functional groups, as well as by counting H-bonds between peptides and lipids. Since K5F2 at 30 °C did not adsorb, it is excluded from this analysis. For the H-bonds investigation, only the period when a peptide was adsorbed was examined. As seen from the RDFs of the peptides (Figure S7 in Supporting Information) around PO_2_^−^, C=O and N(CH_3_)_3_^+^ groups, the shape of the curves remains similar regardless of the peptide in question, meaning their preferred distance is similar and likely governed by H-bonding (in the case of PO_2_^−^ and C=O) or electrostatic repulsion (N(CH_3_)_3_^+^). However, the intensity of the functions is significantly larger for R5F2, meaning that the probability of interaction is higher. This is also reflected in the average number of H-bonds established between peptides and lipid groups. On average, R5F2 established in total 8 ± 2 and 11 ± 3 H-bonds with lipids at 30 °C and 50 °C, respectively, while K5F2 only formed 5 ± 2 H-bonds at 50 °C. The majority of those bonds were made with the PO_2_^−^ group (9 ± 2 for R5F2 vs. 4 ± 2 for K5F2 at 50 °C), while C=O H-bonds are sparse (2–3 for R5F2 or 1–2 for K5F2). Thus, it is shown that both peptides, when adsorbed, are inserted among the lipid headgroups and may easily interact with the phosphate group and occasionally contact the carboxyl. The attraction of cationic peptide components with the negatively charged phosphate was expected, but the discrepancy in H-bonding is due to amino acid-specific effects. Guanidine groups of Arg have more potential H-bond donors compared to amine in Lys, and their planar shape and delocalized charge distribution are particularly conducive to binding lipid groups [14,21,50,51,52]. In particular, the ability of Arg residues to simultaneously bind both phosphate and carboxyl lipid groups was seen as the important cause of different membrane behavior [51,53]. The stronger potential for H-bonding is one of the important contributors to the larger lipid affinity for Arg-containing peptides.

Although peptides are able to incorporate themselves among lipid headgroups, their strong electrostatic interactions and steric disruptions due to their size likely impede their ability to penetrate further. Importantly, in both peptides the non-polar Phe remained in contact with water rather than being incorporated among the hydrophobic acyl chains, as is known to happen for similar peptides [49,54]. There could be multiple reasons for this effect, from steric hindrance to the nature of the simulation setup, since the size of the box is limited and periodic boundary conditions are in place, requiring lipid molecules to be compressed for peptide insertion [52]. The rearrangement of both peptide conformation and bilayer structure, as well as the penalty for introducing charge into a non-polar environment, likely make the energy barrier for insertion high, so that it would not manifest in the short time of the simulation run [50,52]. 

The impact of peptide binding on the membrane can also be investigated by examining structural parameters such as area per lipid (APL) and membrane thickness. APL was calculated from the membrane surface area (given by the product of the *x* and *y* dimensions of the simulation box) divided by the number of lipids in one leaflet. Membrane thickness was obtained from the distance between the peak maxima in the density profile of the phosphorus atom. The values listed in Table 2 are consistent with the reports for DPPC membranes in the gel and fluid phases [55,56], as well as our previous calculations in the absence of peptides [21,27]. Since K5F2 was predominantly found in solvent and only sporadically interacted with the membrane, the sections of simulation trajectories where K5F2 was unbound (200–300 ns) can be used to assess the membrane structure in the absence of peptides. A comparison of K5F2 systems where APL and thickness were taken from whole or partial trajectories yielded no significant difference, showing the negligible impact of K5F2 adsorption or transitory interactions. Therefore, the discussion focus will be placed on the differences present in R5F2 systems, which had an adsorbed period for the full duration of the studied window.

In the gel phase, the presence of R5F2 leads to a decrease of APL coupled with an increase in thickness. In the fluid phase, there is no change in APL regardless of peptide type, but the thickness is still increased in the system containing R5F2. Considering that the R5F2 adsorbs to the membrane at both temperatures and remains attached, its insertion among membrane headgroups is likely the cause of structural re-arrangement. The interaction with Arg residues results in bridging the neighboring lipid headgroups by multiple H-bonds [51], allowing for better ordering. The increase in ordering is also visible from acyl chain order parameters (see Figure S8 and the corresponding discussion in Supporting Information). Interestingly, some literature reports [53,57] claim that certain Arg-peptides induce the exact opposite membrane changes upon adsorption, which would also be more in line with some of our experimental results (see discussion on FTIR data). However, their research setup either involved negatively charged lipids or multiple peptides in the simulation box, both of which are not present in our simulations. It may be that the discrepancy between our simulations and experimental setups in terms of the number of peptides and the presence of 5% DPPG impacts the conclusions on bilayer organization. Even so, here the obtained structural parameters for R5F2 systems in the gel phase would indicate slightly decreased membrane fluidity, which would be in accordance with a small increase in the transition temperatures seen from DSC, but contrary to UV-Vis measurements. 

Regardless of the contradictions obtained from DSC and UV-Vis, the presented experimental data suggest that the principal difference in the adsorption of R5F2 and K5F2 on the DPPC′ surface is associated with the presence/absence of H-atom transfer from titratable functional groups of R5F2/K5F2 [21] and the different impact of adsorbed peptides on van der Waals interactions between hydrocarbon chains and lateral packing.

**Figure 5:**
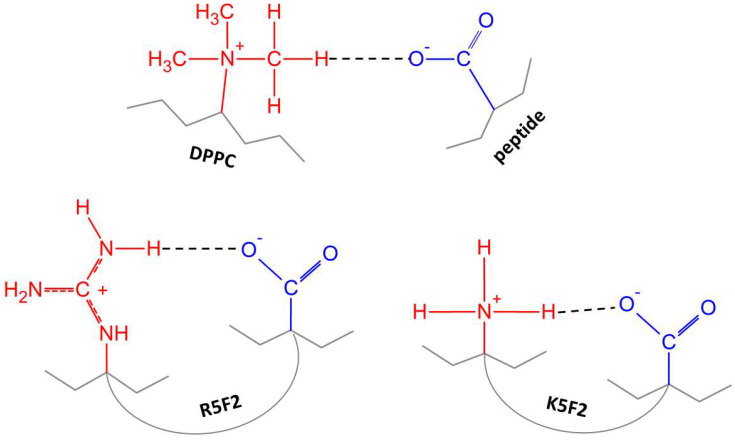
The illustration of possible interactions between the carboxyl group of peptides and DPPC (**top**) or cationic residues within peptide side chains (**bottom**). Hydrogen donors are marked in red, and hydrogen acceptors in blue.

As the classical MD simulation procedure applied here did not allow for the modeling of covalent bond breaking/formation, the proton transfer could not be confirmed computationally. Though some procedures for the implementation of proton transfer calculations exist, they would incur significant computational cost and require parametrization for membrane-peptide systems [58]. From FTIR experiments, though, it seems most likely that the adsorption of R5F2 on the DPPC′ surface is accompanied by a peptide conformational change that makes it capable of either intramolecular proton transfer (COO^−^∙∙∙H_2_N^+^=) or proton exchange with the choline group of DPPC lipids (Figure 5). Both scenarios result in the appearance of the COOH group and the (almost) vanishing of the signal originated from ν_s_COO^−^ (Figure 3e), which is opposite to the situation observed when R5F2 is dissolved in PB (see Figures S3 and S4). As the spectral appearances are reversed when K5F2 is adsorbed on the DPPC surface or dissolved in PB, we are inclined to assume that the interaction of COO^−^ moiety of K5F2 with DPPC′ (see Figure 3b,f) governs the adsorption, while this phenomenon in an intramolecular fashion (COO^−^∙∙∙H_3_N^+^) very likely occurs when K5F2 is dissolved in PB (see FTIR spectra in Figures S3 and S4) [59]. These proton transfer-related opposites might be mirrored in different trends of phase transition temperatures (*T*_pt_) of DPPC′ when interacting with R5F2/K5F2 (see Table 1), and a more detailed explanation of the observed phenomena will be provided in a subsequent work. 

Ultimately, the findings provided in this research should not be limited only to the context of Arg- and Lys-rich peptides as CPPs; they may also open the way of understanding the mode of action of intrinsically neuroprotective Arg-rich peptides [60,61,62] and their antifungal potential [63], while their fundamental differences in adsorption/desorption processes might help to explain the stronger antiviral activity of Arg-rich compared to Lys-rich peptides [64,65].

## 5. Conclusions

In this manuscript, we examined the adsorption mechanism of reported cell-penetrating peptides on DPPC′ lipid bilayers. The adsorption of R5F2/K5F2 on DPPC′ is reflected in the alternations of DPPC′ thermotropic properties, but calorimetric and spectrophotometric data were demonstrated to be inconsistent and, at this very moment, it does not seem possible to state whether peptide adsorption contributes to the rigidity or softening of lipid bilayers. From a molecular-level point of view, it is unequivocally demonstrated that when R5F2 is adsorbed on the DPPC′ surface, a proton transfer that encompasses carboxylic moiety occurs, whereas the reverse scenario appears when K5F2 is adsorbed on the DPPC′ surface. MD simulations demonstrate the differing ad(de)sorption behavior of K5F2 and R5F2 stemming from the different ability of cationic amino acids to establish H-bonds and other interactions with lipid headgroups. Thus, R5F2 shows a strong preference for attaching to the lipid bilayer, while K5F2 desorbs easily and spends more time in the solvent phase. Ultimately, in the context of cell-penetrating peptides that are able to carry the cargo and deliver it inside the cell, one has to have in mind that proton transfer, being the inherent property of the hereby investigated cell-penetrating peptides, clearly highly depends on the immediate surroundings. More importantly, by varying the membrane composition one might tune its translocation ability and thus indirectly regulate the efficiency of cargo delivery. Future research related to this issue is underway. 

## Figures and Tables

**Table 1:** Temperatures of the phase transitions (*T*_pt_) of DPPC′ in the presence (and absence) of R5F2/K5F2 determined from the maxima of DSC curves (*T*_p, m_ and *T*_m, m_ for pre- and main-phase transition, respectively) and from inflection points of concentration profiles obtained from multivariate curve analysis (MCA; *T*_p_ and *T*_m_ for pre- and the main phase transition, respectively).

System	*T*_pt_ ^a^			
	DSC		UV-Vis	
	~*T*_p, m_	*T*_m, m_	*T*_p_	*T*_m_
DPPC′ + R5F2	34	41.6 ± 0.2	33.6 ± 0.3	42.0 ± 0.4
DPPC′ + K5F2	32	40.5 ± 0.1	36.1 ± 0.7	44.2 ± 0.5
DPPC′	33.2	41.4 ± 0.1	34.4 ± 0.2	42.2 ± 0.1

^a^ In °C.

**Table 2:** Structural parameters of simulated bilayers at two temperatures, in the presence of either K5F2 or R5F2 peptides, as well as the comparison with DPPC alone. The values of DPPC with no peptides attached were taken from K5F2 simulations in the period where no binding occurred (200–300 ns). Also, the initial values of pure DPPC membrane following CHARMM-GUI equilibration, but before peptide addition and full production run, are reported in brackets.

*T* ^a^	Peptide	APL ^b^	Membrane Thickness ^c^
30	K5F2	0.517 ± 0.006	4.940 ± 0.032
	R5F2	0.498 ± 0.005	5.150 ± 0.083
	none	0.518 ± 0.005 (0.544 ± 0.003)	4.924 ± 0.072 (4.054 ± 0.038)
50	K5F2	0.614 ± 0.013	3.905 ± 0.007
	R5F2	0.613 ± 0.012	4.018 ± 0.014
	none	0.615 ± 0.013 (0.600 ± 0.006)	3.905 ± 0.041 (4.022 ± 0.028)

^a^ In °C; ^b^ In nm^2^; ^c^ In nm.

## Data Availability

Not applicable.

## Supplementary Materials

The following supporting information can be downloaded at: https://www.mdpi.com/article/10.3390/antibiotics12071216/s1 [66], Scheme S1: Solid phase synthesis of heptapeptides K5F2 and R5F2; Scheme S2: ^1^H spectra of K5F2 peptide; Scheme S3: ^1^H spectra of R5F2 peptide; Scheme S4: ^13^C spectra of K5F2 peptide; Scheme S5: ^13^C spectra of R5F2 peptide; Scheme S6: HRMS spectra for the K5F2 peptide in *m*/*z* 946–966; Scheme S7: HRMS spectra for the R5F2 peptide in *m*/*z* 1080–1110; Figure S1: DLS data of: a) DPPC; b) DPPC′+R5F2; c) DPPC′+K5F2 obtained at 25 °C; Figure S2: DSC curves of: a) R5F2 and b) K5F2 in PB, respectively, in the temperature range 30–52 °C obtained in the 2nd heating run; Figure S3: FTIR spectra of R5F2/K5F2 in PB in the following spectral ranges: a, b) 3000–2820 cm^−1^ (ν_as_CH_3_, ν_(a)s_CH_2_); c, d) 1770–1700 cm^−1^ (νC=O); e, f) 1515–1360 cm^−1^ (γCH_2_, ν_s_COO^−^). The spectra are labelled as short dotted (0.001 mol dm^−3^) and short dashed (0.01 mol dm^−3^) red/wine (30 °C/50 °C) curves for R5F2 and blue/navy (30 °C/50 °C) for K5F2, respectively. The envelope maxima are designated with yellow lines and yellow rectangles; Figure S4: Spectral range 1800–1475 cm^−1^ that encompasses νC=O and Amide I and Amide II bands of: a) R5F2 and b) K5F2 in PB, respectively, acquired at 30 °C (red/blue curves for R5F2/K5F2) and 50 °C (wine/navy curves for R5F2/K5F2); short dotted curves designate *c*(R5F2/K5F2) = 0.001 mol dm^−1^ and short dashed curves designate *c*(R5F2/K5F2) = 0.01 mol dm^−1^. Envelopes maxima are highlighted with yellow lines. The spectrum of K5F2 (*c*(K5F2) = 0.001 mol dm^−1^) at 30 °C display considerable response of H_2_O molecules (δHOH); Figure S5: FTIR spectra of DPPC′ ± R5F2/K5F2 in the spectral range 1275–1190 cm^−1^. DPPC′ spectra are presented with solid gray/dark gray (30 °C/50 °C) curves, DPPC′ + R5F2 with solid red/wine (30 °C/50 °C) curves, and DPPC′ + K5F2 with solid blue/navy (30 °C/50 °C) curves. The spectra of R5F2/K5F2 are labeled with the same color as corresponding spectra of DPPC′ + R5F2/K5F2 but with dotted curves. Along with the band assignment, their displacement in DPPC′ ± R5F2/K5F2 are designated with light gray-shaded rectangles or solid light gray lines; Figure S6: S6 Number density distributions of select components of the simulation box along *z*-axis: a) K5R2 at 30 °C, b) R5F2 at 30 °C, c) K5R2 at 50 °C and d) R5F2 at 50 °C. The position of the membrane is denoted by two peaks belonging to phosphorus atoms; Figure S7: Radial distribution functions of Arg or Lys around PO_2_^−^, C=O and N(CH_3_)_3_^+^ groups in examined systems; Figure S8: Deuterium order parameters of carbon atoms in DPPC acyl chains for the examined systems. The average of both *sn*-1 and *sn*-2 chains is reported.
