# Supplementary material for: Adsorption/Desorption of Cationic-Hydrophobic Peptides on Zwitterionic Lipid Bilayer Is Associated with the Possibility of Proton Transfer
Source: Antibiotics (Basel). 2023 Jul 21;12(7):1216. doi: 10.3390/antibiotics12071216 (PMC10376034; doi:10.3390/antibiotics12071216)
Supplement: Supplementary file 1 [file antibiotics-12-01216-s001.zip › antibiotics-2513694-supplementary.pdf]

Adsorption/desorption of cationic-hydrophobic peptides on zwitterionic lipid bilayer is associated with the possibility of proton transfer

Lea Pašalić, Andreja Jakas, Barbara Pem, Danijela Bakarić*

Division of Organic Chemistry and Biochemistry, Ruđer Bošković Institute, Bijenička cesta 54, 10000 Zagreb, Croatia

*Corresponding author. Tel.: +385 1 4571 382; E-mail: danijela.bakaric@irb.hr (Danijela Bakarić)

Supporting Information

S1. R5F2/K5F2: solid-phase synthesis description and spectral characterization p1

S2. DLS data of DPPC′ ± R5F2/K5F2 p9

S3. DSC curves of R5F2/K5F2 in PB p10

S4. FTIR data of R5F2/K5F2 at c(R5F2/K5F2) = 0.001 mol dm^{-3} and 0.01 mol dm^{-3} p11

S5. Additional FTIR data of DPPC′ ± R5F2/K5F2 p14

S6. Additional molecular dynamics data p15

References p18

S1. R5F2/K5F2: solid-phase synthesis description and spectral characterization

General methods:

N-methylmorpholine (NMM) was purchased from Merck. Fmoc-protected amino acids, HBTU, HOBt, and Fmoc-protected amino acids-Wang resin (0.56 mmol/g; 150 mg, 0.084 mmol), were purchased from Novabiochem.

The solid support synthesis was preformed automatically on PS3 Bio-Organic synthesizer (PROTEIN TECHNOLOGIES Inc, Tucson, Arizona, USA.) using Fmoc-protected amino acids, condensing reagents HBTU and HOBt, with base NMM. For Fmoc-deprotection 20% piperidine/DMF was used, and for side chain deprotection and cleavage from resin TFA:TIS:H_2O 95:2.5:2.5. [1]

Peptides were purified on RPHPLC 1260 Infinity II Agilent Technologies on column ZORBAX Extend-C18 Prep. (5 µm, 21.2 × 150 mm, flow 10 mL/min). Products purity was monitored on RPHPLC 1200 Series Agilent Technologies on analytical column ZORBAX Extend-C18 (5 µm, 4.6 × 250 mm, flow 1.0 mL/min), at wavelengths 215 and 254 nm. Following solvents were used for HPLC: A Gradient: 0 → 30 min, 100% 0.1% TFA → 100% MeOH ; 30 → 35 min, 100% MeOH; 35 → 35.01 min, 100% MeOH → 0.1% TFA; 35.01 → 40 min, 100% 0.1% TFA.

NMR spectra were recorded on a Bruker AV 600 spectrometer, operating at 150.91 MHz for ^{13}C and 600.13 MHz for ^{1}H nuclei. The spectra were measured in DMSO-d_6 at 25 °C. Chemical shifts in parts per million (ppm) were referenced to tetramethylsilane (TMS). Spectra were assigned based on one dimensional ^{1}H, ^{13}C and APT (Attached Proton Test) and 2D homonuclear COSY (Correlation Spectroscopy) and heteronuclear HMQC (Heteronuclear Multiple-Quantum Correlation) and HMBC (Heteronuclear Multiple Bond Correlation) experiments.

High resolution accurate mass data were obtained on Agilent 6546 LC/Q-TOF spectrometer (Santa Clara, CA, USA) under electrospray ionization technique. Samples were dissolved in 0.1% formic acid to the concentration of 0.1 mg/mL and placed in the multisampler for analysis using the 1290 Infinity II LC and 6546 LC/Q-TOF with Jet Stream source. Purine (m/z 121.050873) and HP-921 (hexakis (1H,1H,3H-tetrafluoropropoxy) phosphazene, m/z 922.009798) were used as reference ions during the analysis to achieve the best mass accuracy.

Peptide synthesis:

The solid support synthesis was preformed automatically on PS3 Bio-Organic synthesizer (PROTEIN TECHNOLOGIES Inc, Tucson, Arizona, USA) using Fmoc-protected amino acids, condensing reagents HBTU and HOBt, with base *N*-methylmorpholine (NMM) [1]. NMM was purchased from Merck. Fmoc-protected amino acids, HBTU, HOBt, and Fmoc-protected amino acids-Wang resin (0.56 mmol/g; 150 mg, 0.084 mmol), were purchased from Novabiochem. 250 mg (0.1 mmol) Fmoc-Phe-Wang resin was placed in instrument (PS3) reaction vessel and program for swelling and deprotection (20% piperidine/DMF) was run. Second amino acid 155 mg (0.4 mmol) Fmoc-Phe-OH was activated in the instrument vial with HBTU (54 mg; 0.4 mmol) and HOBt (152 mg; 0.4 mmol) in DMF and sucked to reaction vessel. Coupling was continued for 1 hour followed with deprotection of Fmoc protecting group. Process of coupling/deprotection (HBTU,HOBt/20% piperidine) was repeated for every following amino acid, and finished with Fmoc deprotection of final amino acid Lys/Arg. Dry resin was transferred to PVC syringe and deprotected with TFA:TIS:H_2O 95:2.5:2.5 for 3 hours. The solution was dropped to cuvette filed with diisopropylether and lived overnight in refrigerator. Precipitate was centrifuged and washed 2x with didisopropylether, and dried. Crude product was purified on preparative HPLC on reversed phase column in system A. Purity was check on analytical RPHPLC.

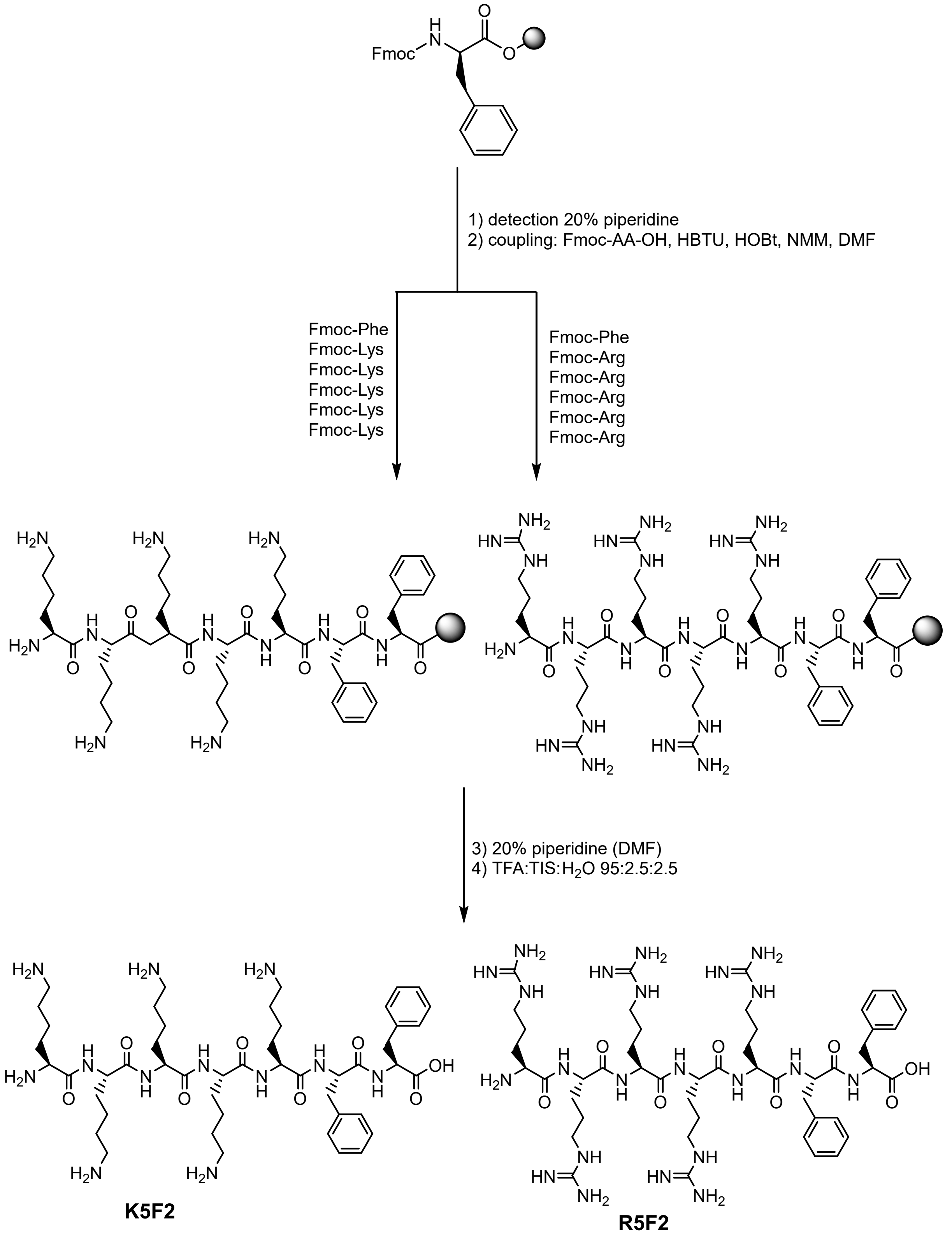

Scheme 1. Solid phase synthesis of heptapeptides K5F2 and R5F2

Characterization of K5F2 and R5F2 peptides

*L-lysyl-L-lysyl-L-lysyl-L-lysyl-L-lysyl-L-phenylalanyl-L-phenylalanine (KKKKKFF, **K5F2**)*

Yield: 80%; *t*R (A) = 17.1. 1H NMR(DMSO-d_6) *δ*/ppm: 8.59-7.96 NH (d, 6H), 7.90-7.85 NH_2 (m, 12H), 7.28-7.17 CH Ar-$Phe^{6,7}$-δ,ε.ζ (m, 10H), 4.55-4.52 Phe^{6}-α (m, 1H), 4.47-4.44 Lys^{2}-α (m, 1H), 4.31-4.28 Phe^{7}-α (m, 1H), 4.24-4.13 $Lys^{1,3,5}$- α (m, 4H), 3.08-2.91 $Phe^{6,7}$-β (m, 4H), 2.77-2.68 Lys^{1-5}-ε (m, 10H), 1.76-1.20 $Lys^{1,5}$-δ, $Lys^{1,5}$-β, Lys^{1-5}-γ (m, 30H).

13C NMR(DMSO-d_6) δ/ppm: [172.6, 171.4, 171.2, 171.1, 171.0, 168.4] CO, [137.4, 137.3] $Phe^{6,7}$-γ, [129.2, 129.1, 128.2, 127.9, 126.4, 126.2] Phe-δ, Phe-ε, Phe- ζ, [53.4, 53.3] $Phe^{6,7}$-α, [52.5, 52.3, 51.8] Lys^{1-5}-α, [38.6, 38.4] Lys^{1-5}-ε, [31.3, 30.4] Lys^{1-5}-δ, [26.6, 26.3] Lys^{1-5}-β, [22.2, 22.1, 21.0] Lys^{1-5}-γ. $C_{48}H_{80}N_{12}O_8$ *M*r = 952.62, $[M + H]^+$. HRMS: *m/z* 953,6298 $[M + H]^+$ (calc. 953,6300).

*L-arginyl-L-arginyl-L-arginyl-L-arginyl-L-arginyl-L-phenylalanyl-L-phenylalanine (RRRRRFF, **R5F2**)*

Yield: 76 %; *t*R (A) = 17.2. 1H NMR(DMSO-d_6) *δ*/ppm: 8.61–7.65 NH (m, 14H), 7.23-7.15 Phe-δ, Phe-ε, Phe-ζ-, NH, NH_2 (m, 26H), 4.56-4.53 Phe^{6}-α (m, 1H), 4-46-4.43 Arg^{2}-α (m, 1H), 4.35-4.32 α-Phe^{7}-α (m, 1H), 4.25-4.18 Arg^{3-5} -α (m, 3H), 3.85-3.82 Arg^{1}-α (m, 1H), 3.11-2.99 $Arg^{1,5}$-δ (m, 12H), 2.98-2.90 $Phe^{6,7}$-β (dd, 2H), 2.77-2.73 β'-$Phe^{6,7}$- β' (dd, 2H), 1.69-1.35 Arg^{1-5}-β, Arg^{1-5}-γ (m, 20H).

13C NMR(DMSO-d_6) δ/ppm: [172.7, 171.3, 171.1, 171.0, 170.9, 168.4] CO, [159.4, 159.1, 158.9, 158.7, 156.9, 156.9, 156.8] Arg^{1-5}-δ, [137.3] $Phe^{6,7}$-γ, [129.2, 129.1, 128,2,128.0, 126.4, 126.2] $Phe^{6,7}$-δ, $Phe^{6,7}$-ε, $Phe^{6,7}$-ζ, [53.5, 53.4] α-$Phe^{6,7}$-α, [52.4, 52.2, 52.1,51.7] Arg^{1-5}-α, [40.4, 40.1, 40.0] Arg^{1-5}-δ, [37.6, 36.7] $Phe^{6,7}$-β, [29.2, 28.4] Arg^{1-5}-β, [25.0, 24.9, 24.8, 24.0] Arg^{1-5}-γ.

$C_{48}H_{80}N_{22}O_8$ *M*r = 1093.66, $[M + H]^+$. HRMS: *m/z* 1093,6608 $[M + H]^+$ (calc. 1093,6608).

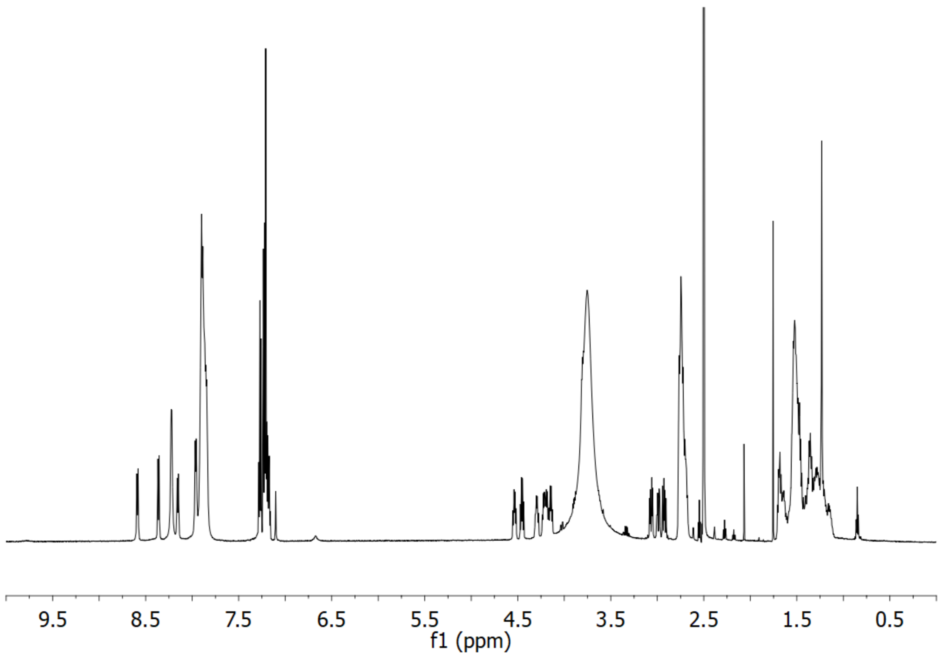

Scheme 2. ^{1}H spectra of K5F2 peptide

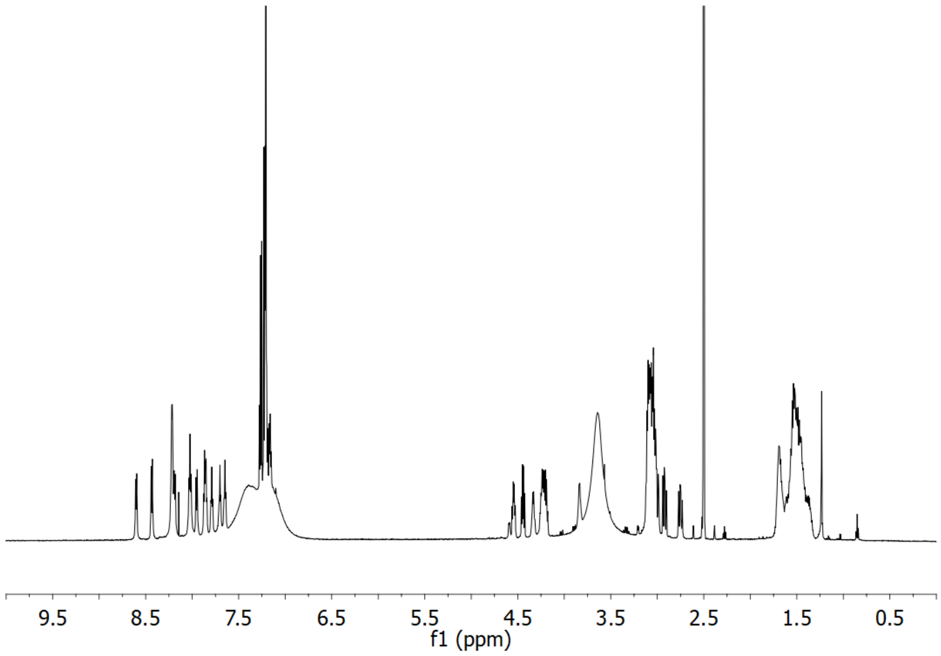

Scheme 3. ^{1}H spectra of R5F2 peptide

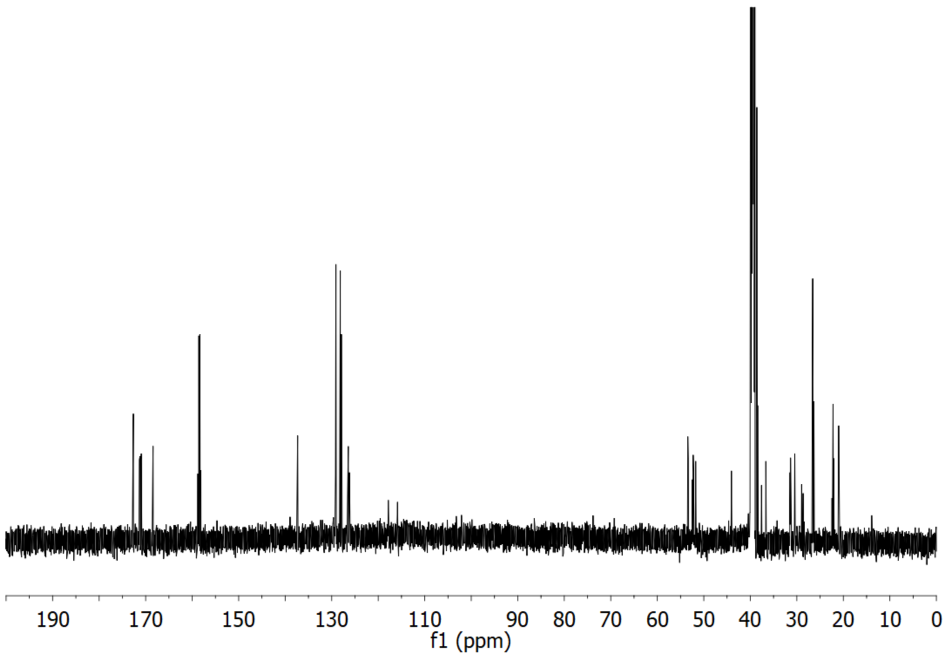

Scheme 4. ^{13}C spectra of K5F2 peptide

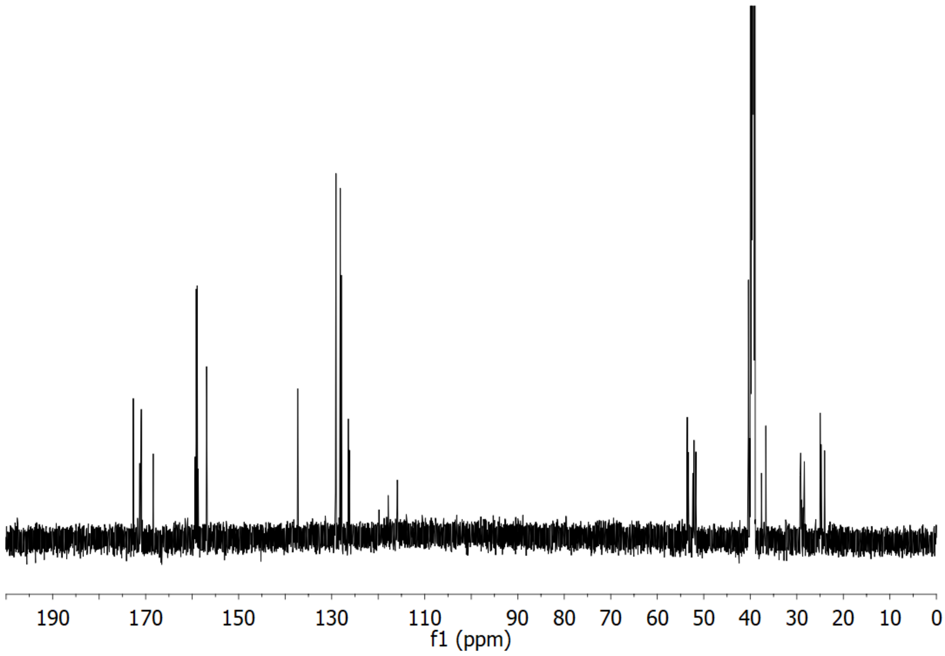

Scheme 5. ^{13}C spectra of R5F2 peptide

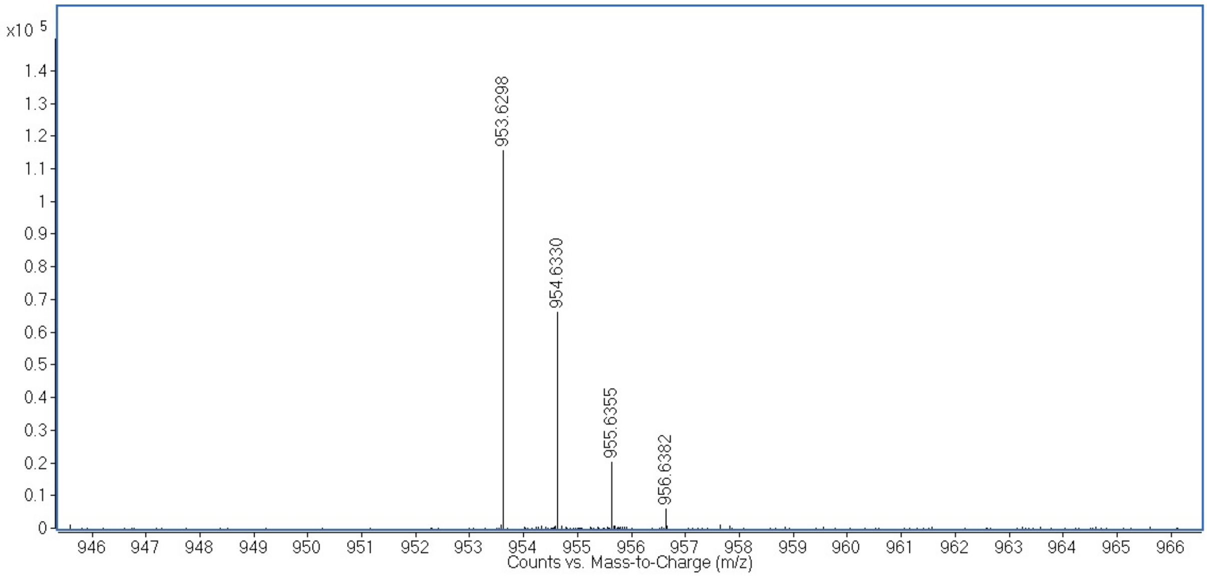

Scheme 6. HRMS spectra for the K5F2 peptide in m/z 946 – 966

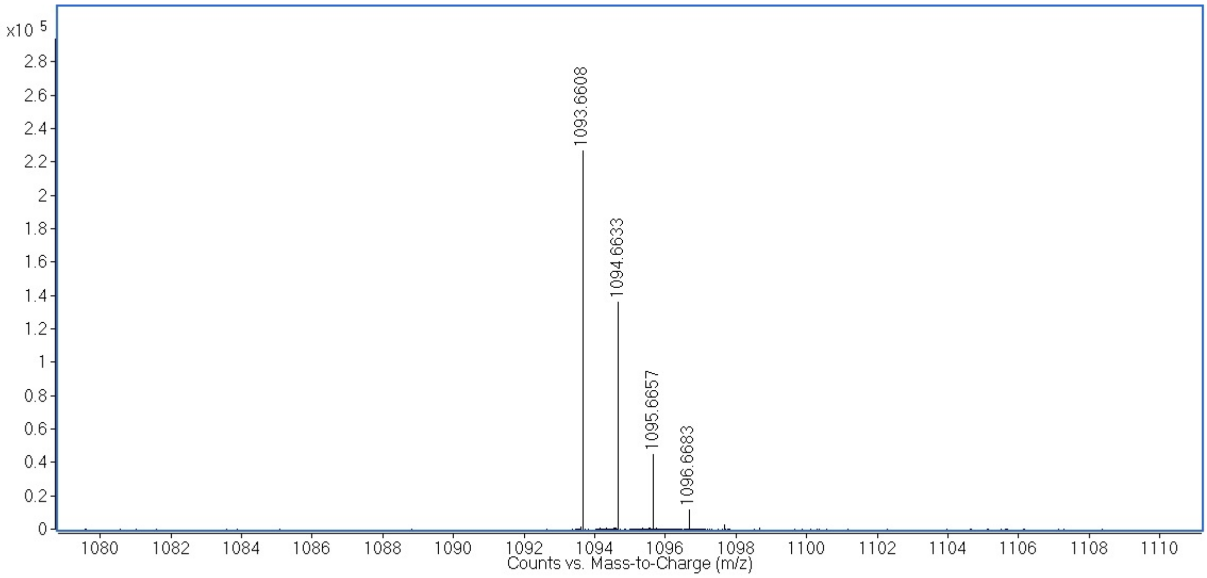

Scheme 7. HRMS spectra for the R5F2 peptide in m/z 1080 – 1110

S2. DLS data of DPPC’ ± R5F2/K5F2

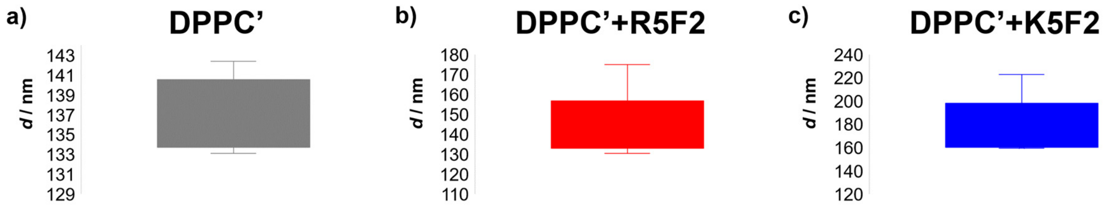

Fig. S1. DLS data of: a) DPPC; b) DPPC″+R5F2; c) DPPC′+K5F2 obtained at 25 °C.

S3. DSC curves of R5F2/K5F2 in PB

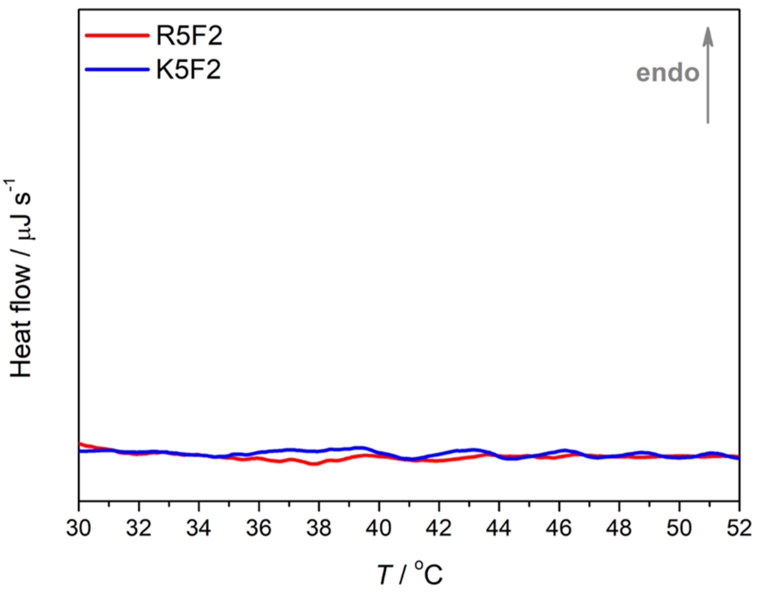

Fig. S2. DSC curves of: a) R5F2 and b) K5F2 in PB, respectively, in the temperature range 30-52 °C obtained in the 2nd heating run.

S4. FTIR data of R5F2/K5F2 at *c*(R5F2/K5F2) = 0.001 mol dm^{-3} and 0.01 mol dm^{-3}

FTIR spectra of R5F2/K5F2 having *c*(R5F2/K5F2) = 0.001 mol dm^{-3} and 0.01 mol dm^{-3} subtracted from the spectrum of PB are presented in Fig. S3. The most distinguished bands are those originated from $\nu_{(a)s}CH_2$ (at 2925 cm^{-1} and at 2854 cm^{-1}) (Fig. S3a, b), νC=O (at 1744 cm^{-1}) (Fig. S3c, d) and γCH_2 and $\nu_s COO^-$ (in the range of about 1480-1420 cm^{-1} and 1420-1360 cm^{-1}) (Fig. S3e, f).

Fig. S4 presents the spectral range 1800-1475 cm^{-1} of R5F2/K5F2 that, along νC=O (with maxima at 1744 cm^{-1}/1744 cm^{-1}), contains the signals of Amide I and Amide II bands (with maxima at 1678 cm^{-1}/1678 cm^{-1}, 1632 cm^{-1}/1639 cm^{-1} and 1548 cm^{-1}/1533 cm^{-1}) as well as the contribution of H_2O that is especially pronounced in FTIR spectrum of K5F2 with *c*(K5F2) = 0.001 mol dm^{-3} (maximum at about 1640 cm^{-1}).

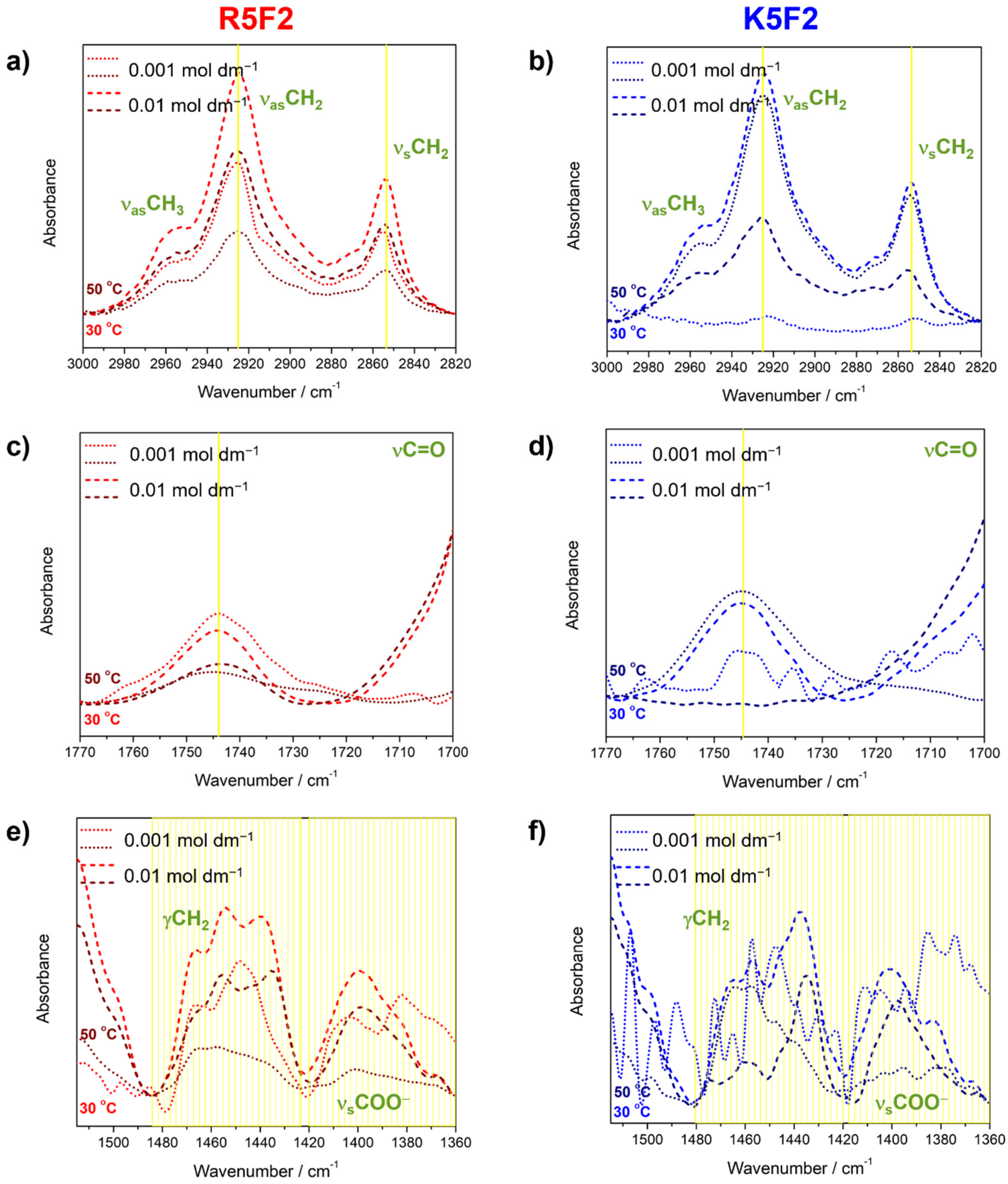

Fig. S3. FTIR spectra of R5F2/K5F2 in PB in the following spectral ranges: a, b) 3000-2820 cm^{-1} ($\nu_{as}CH_3$, $\nu_{(a)s}CH_2$); c, d) 1770-1700 cm^{-1} ($\nu C{=}O$); e, f) 1515-1360 cm^{-1} (γCH_2, $\nu_s COO^-$). The spectra are labelled as short dotted (0.001 mol dm^{-3}) and short dashed (0.01 mol dm^{-3}) red/wine (30 °C/50 °C) curves for R5F2 and blue/navy (30 °C/50 °C) for K5F2, respectively. The envelope maxima are designated with yellow lines and yellow rectangles.

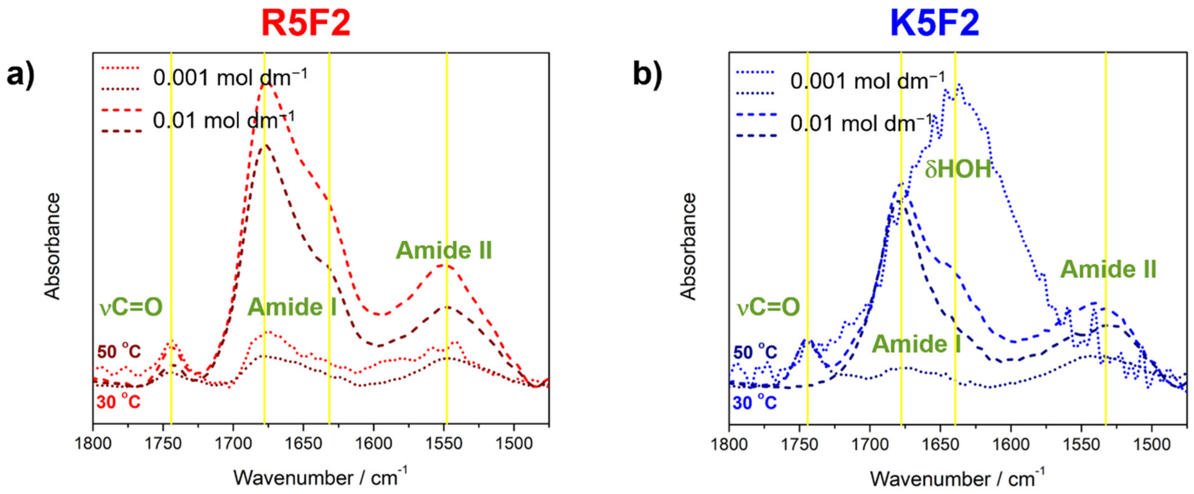

Fig. S4. Spectral range 1800-1475 cm^{-1} that encompasses νC=O and Amide I and Amide II bands of: a) R5F2 and b) K5F2 in PB, respectively, acquired at 30 °C (red/blue curves for R5F2/K5F2) and 50 °C (wine/navy curves for R5F2/K5F2); short dotted curves designate *c*(R5F2/K5F2) = 0.001 mol dm^{-1} and short dashed curves designate *c*(R5F2/K5F2) = 0.01 mol dm^{-1}. Envelopes maxima are highlighted with yellow lines. The spectrum of K5F2 (*c*(K5F2) = 0.001 mol dm^{-1}) at 30 °C display considerable response of H_2O molecules (δHOH).

S5. Additional FTIR data of DPPC′ ± R5F2/K5F2

The spectral region presented in Fig. S4 dominantly presents the signals originated from the antisymmetric stretching of phosphate groups ($\nu_{as}PO_2^-$), both hydrogen-bonded (HB) and non-hydrogen-bonded (non-HB) [2]. In particular, the envelope maximum is observed at 1229 cm^{-1}/1232 cm^{-1} at 30 °C/50 °C in DPPC′, at 1229 cm^{-1}/1232 cm^{-1} at 30 °C/50 °C in DPPC′+R5F2 and at 1232 cm^{-1}/1232 cm^{-1} at 30 °C/50 °C in DPPC′+K5F2 (additionally, the envelope is more structured in the last system.

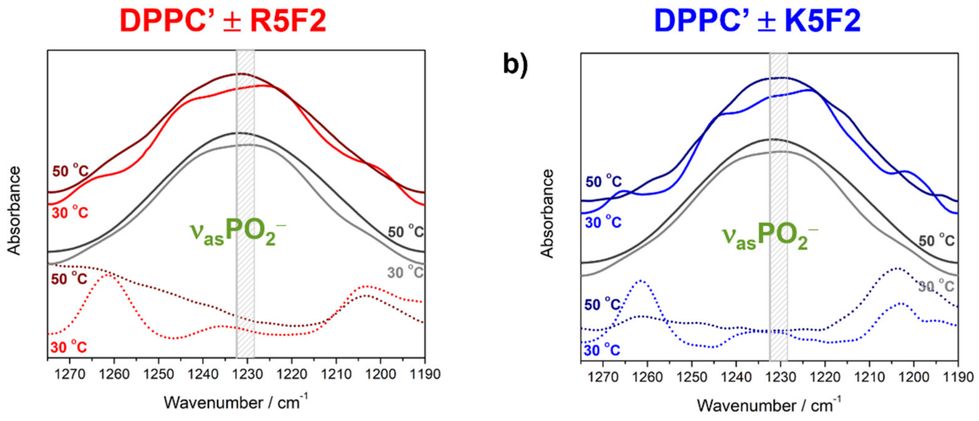

Fig. S5. FTIR spectra of DPPC′ ± R5F2/K5F2 in the spectral range 1275-1190 cm^{-1}. DPPC′ spectra are presented with solid gray/dark gray (30 °C/50 °C) curves, DPPC′ + R5F2 with solid red/wine (30 °C/50 °C) curves, and DPPC′ + K5F2 with solid blue/navy (30 °C/50 °C) curves. The spectra of R5F2/K5F2 are labeled with the same color as corresponding spectra of DPPC′ + R5F2/K5F2 but with dotted curves. Along with the band assignment, their displacement in DPPC′ ± R5F2/K5F2 are designated with light gray-shaded rectangles or solid light gray lines.

S6. Additional molecular dynamics data

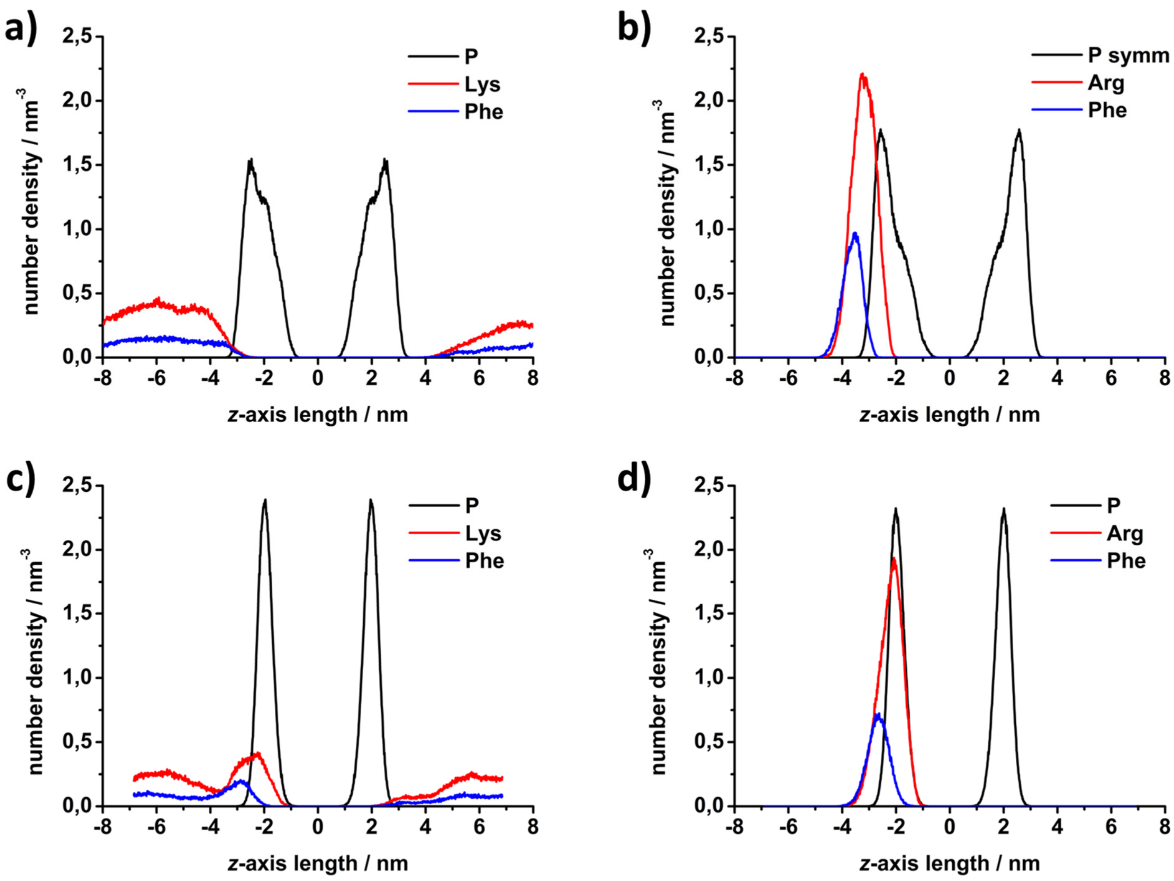

Fig. S6. Number density distributions of select components of the simulation box along *z*-axis: a) K5R2 at 30 °C, b) R5F2 at 30 °C, c) K5R2 at 50 °C and d) R5F2 at 50 °C. The position of the membrane is denoted by two peaks belonging to phosphorus atoms.

The number density distributions demonstrate the average position of peptide residues in relation to the membrane, during the final 200 ns of production. In the case of K5F2, its presence in the solvent is always seen, but at 50 °C it is also located partially on the membrane. R5F2 is found exclusively on the membrane surface, regardless of temperature. Arg/Lys are always positioned closer to the membrane, and Phe away towards water. Even so, there is always partial overlap between Phe and phosphorus, indicating that Phe can be found among the lipid headgroups at least some of the time.

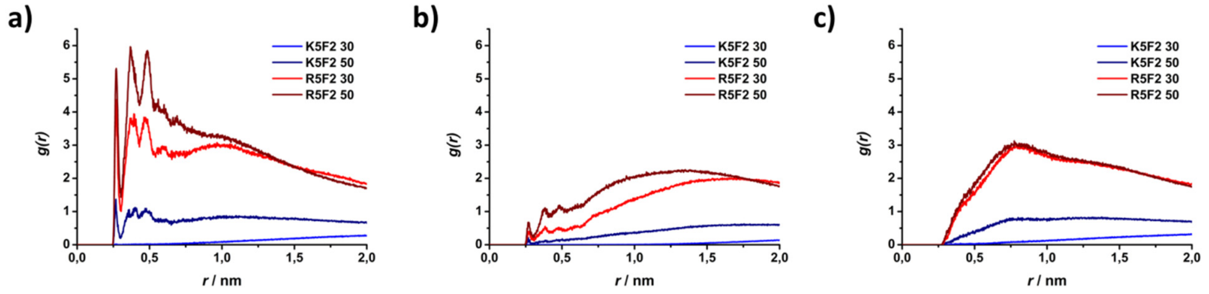

Fig. S7. Radial distribution functions of Arg or Lys around PO_2^-, C=O and $N(CH_3)_3^+$ groups in examined systems.

RDFs represent the probability of locating the examined atom or group at a certain distance from a reference atom or a group. In this case, R5F2 and K5F2 position (excluding hydrogen atoms) was examined in relation to main lipid functional groups. RDFs of peptides around PO_2^- show maxima at 0.27 nm, 0.37 nm and 0.49 nm, which correspond to the distance between the H-bond donor of the peptide and acceptor oxygen, carbon and other oxygen of the phosphate group, respectively. The same maximum at 0.27 nm is seen in RDFs with C=O group as a reference, since it also establishes a H-bond, but its intensity is much lower since those bonds are rare. RDFs of the $N(CH_3)_3^+$ groups of choline have a very broad peak with a maximum around 0.77 nm, because of the repulsive forces of the positively charged groups.

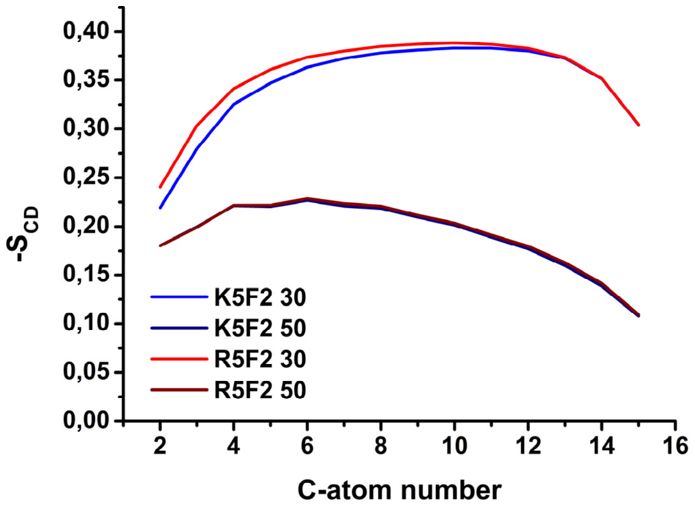

Fig. S8. Deuterium order parameters of carbon atoms in DPPC acyl chains for the examined systems. The average of both *sn*-1 and *sn*-2 chains is reported.

Deuterium order parameters ($-S_{CD}$) are a measure of the level of ordering of lipid acyl chains, where high values point to high level of order (typical for the gel phase, while the inverse is expected for fluid phase). Here, $-S_{CD}$ was calculated by the free software Membrainy [3]. As seen from Fig. S8, the ordering is indeed much higher in the gel phase, while the fluid phase is strongly disordered. In accordance with the APL and membrane thickness results, R5K2 binding does increase lipid ordering, in particular for the upper part of the acyl chain. In fluid phase, the effect is absent, possibly because both peptides are adsorbed for a significant portion of the time, or because the fluid phase membrane is resistant to any structuring influences.

References:

1. Cudic, M.; Fields, G.B. Solid-Phase Peptide Synthesis. In *Molecular Biomethods Handbook*; Walker, J.M., Rapley, R., Eds.; Humana Press: Totowa, NJ; pp. 515–546.

2. Casal, H.L.; Mantsch, H.H. Polymorphic Phase Behaviour of Phospholipid Membranes Studied by Infrared Spectroscopy. *BBA - Rev. Biomembr.* **1984**, *779*, 381–401, doi:10.1016/0304-4157(84)90017-0.

3. Carr, M.; MacPhee, C.E. Membrainy: A "Smart", Unified Membrane Analysis Tool. *Source Code Biol. Med.* **2015**, *10*, 1–10, doi:10.1186/s13029-015-0033-7.
